# Metabolomics and Whole-Exome Sequencing in Patients with Differential Sensitivity to Sevoflurane: A Protocol for a Prospective Observational Trial

**DOI:** 10.3389/fphar.2021.621159

**Published:** 2021-11-01

**Authors:** Yiyong Wei, Donghang Zhang, Yunxia Zuo

**Affiliations:** ^1^ Department of Anesthesiology, West China Hospital of Sichuan University, Chengdu, China; ^2^ Department of Anesthesiology, Affiliated Hospital of Zunyi Medical University, Zunyi, China

**Keywords:** sevoflurane, sensitivity, metabolomics, whole exome sequencing, genome-wide association study

## Abstract

**Introduction:** Different sensitivity to volatile anesthetics in *Drosophila*, nematodes and mice is related to mutation of energy metabolism genes. In clinical practice, we find that the end-tidal sevoflurane concentration (ET_sevo_) differs among patients at the same depth of anesthesia, indicating that the sensitivity to sevoflurane varies among patients. However, the underlying mechanism remains unclear. The sensitivity of an anesthetic is associated with the postoperative outcomes of patients and the mechanism of action of volatile anesthetics. We therefore propose this protocol to determine whether differences in metabolite profile and genetic variations contribute to patients’ sensitivity to volatile anesthetics.

**Methods and Analysis:** This is a single-centre, prospective observational study. 720 patients undergoing abdominal surgery were included. General anesthesia was induced with inhaled sevoflurane, a bolus of sufentanil (0.2–0.4 μg/kg) and *cis*-atracurium (0.2–0.3 mg/kg). The end-tidal sevoflurane concentration (ET_sevo_) was adjusted to maintain a BIS (bispectral index) value between 40–60. The mean ET_sevo_ from 20 min after endotracheal intubation to 2 h after the beginning of surgery (steady state) was calculated for each patient. Patients were further divided into a high-sensitivity group (mean ET_sevo_ – SD) and a low-sensitivity group (mean ET_sevo_ + SD) to investigate the sensitivity to sevoflurane. Cases were paired from the high-sensitivity group (group H) and low-sensitivity group (group L) according to age, sex, body mass index (BMI), *ASA* physical status classification, vital signs, BIS, ephedrine use, sufentanildose, and *cis*-atracurium dose at anesthesia induction and steady state. Differences in metabolite levels, single nucleotide polymorphisms (SNPs) and protein-coding gene sequence variations between group H and group L will be determined through plasma metabolomics, whole-exome sequencing (WES), genome-wide association study (GWAS), and bioinformatics analyses. These results will be analysed to determine the reasons for the differential sensitivity to sevoflurane in humans.

**Ethics and Dissemination:** This prospective observational study protocol has received ethical approval from the Ethical Committee of West China Hospital of Sichuan University on May 19, 2017 (Approval No. 78). Informed consent will be obtained before patient enrolment. The results will be submitted to international peer-review journals.

**Trial Registration Number:** ChiCTR1800014327.

## Introduction

Sevoflurane is one of the most widely used volatile anesthetics, which can produce sedative, analgesic and muscle relaxing effects. Postoperative outcomes of patients may be related to the depth of anesthesia during general anesthesia (Lewis et al., 2019; Liu et al., 2019; Sieber et al., 2019). Studies have shown that excessive anesthetic depth will cause delayed emergence from anesthesia and increase the postoperative mortality of patients (Punjasawadwong et al., 2014; Short et al., 2019), while light anesthesia causes hemodynamic fluctuation, cardiovascular and cerebrovascular adverse events, intraoperative awareness, and disruption of the surgical procedure (Punjasawadwong et al., 2014). In a study including 55 patients with mild depression and major depression, ETsevo was regulated to maintain BIS values between 40 and 60. No difference was found in BIS values between the two groups during cholecystectomy, however, the ETsevo of the major depression group was significantly lower than that of the mild depression group (Erden et al., 2018). This study indicated that the sevoflurane sensitivity may be different in patients (Erden et al., 2018).

Metabolic status can be influenced by general anesthetics, and mitochondrial function may partly contribute to the mechanism of general anesthesia (Yamakage, 2014; Ramadasan-Nair et al., 2017; Zimin et al., 2018). However, the exact effects of general anesthesia on systemic metabolites in humans remain unclear, which may be important for postoperative outcomes. It is widely known that general anesthetics can affect some important metabolism and metabolic connectivity (Alkire et al., 2008; Chen et al., 2019). Sevoflurane inhibits insulin secretion and is associated with hyperglycaemia (Li et al., 2014; Hoyer et al., 2018). However, the exact modulation of systemic metabolism by general anesthetics in humans has not been clearly illustrated. Glutamate is one of the main excitatory neurotransmitters in the brain and is involved in the mechanism of action of inhaled anesthetics (Aksenov et al., 2019). Glutamate transporter 3 (EAAT3) can absorb extracellular glutamate. EAAT3 gene knockout mice exhibit significantly increased sensitivity to isoflurane, indicating that glutamate metabolism may be related to the sensitivity to inhaled anesthetics (Lee et al., 2010).

Mutations of several genes are associated with altered sensitivity to volatile anesthetics in fruit flies, nematodes and mice (Campbell et al., 2009; Nagasaka et al., 2017; Awal et al., 2020). The product of Gas-1 gene is a subunit of mitochondrial complex I (Kayser et al., 2004). The sensitivity to fluorine significantly increased in nematodes with Gas-1 gene mutation, probably due to the inhibition of mitochondrial respiratory function (Kayser et al., 2004). The literature showed that the MAC value and EC50 for isoflurane anesthesia were decreased in shank-3 mutant mice, which may be associated with the decreased expression of NR1 and PSD95 genes (Li et al., 2017).

This study aims to determine differential metabolites, SNPs and genes related to sevoflurane sensitivity among patients through plasma metabolomics, WES, GWAS and bioinformatics. Therefore, this study may identify potential markers of sevoflurane sensitivity for further studies.

## Methods and Analysis

Detailed methods with regards to the intervention and analysis are available in the full pilot study protocol included with this summary.

### Study Design

**FIGURE 1:**
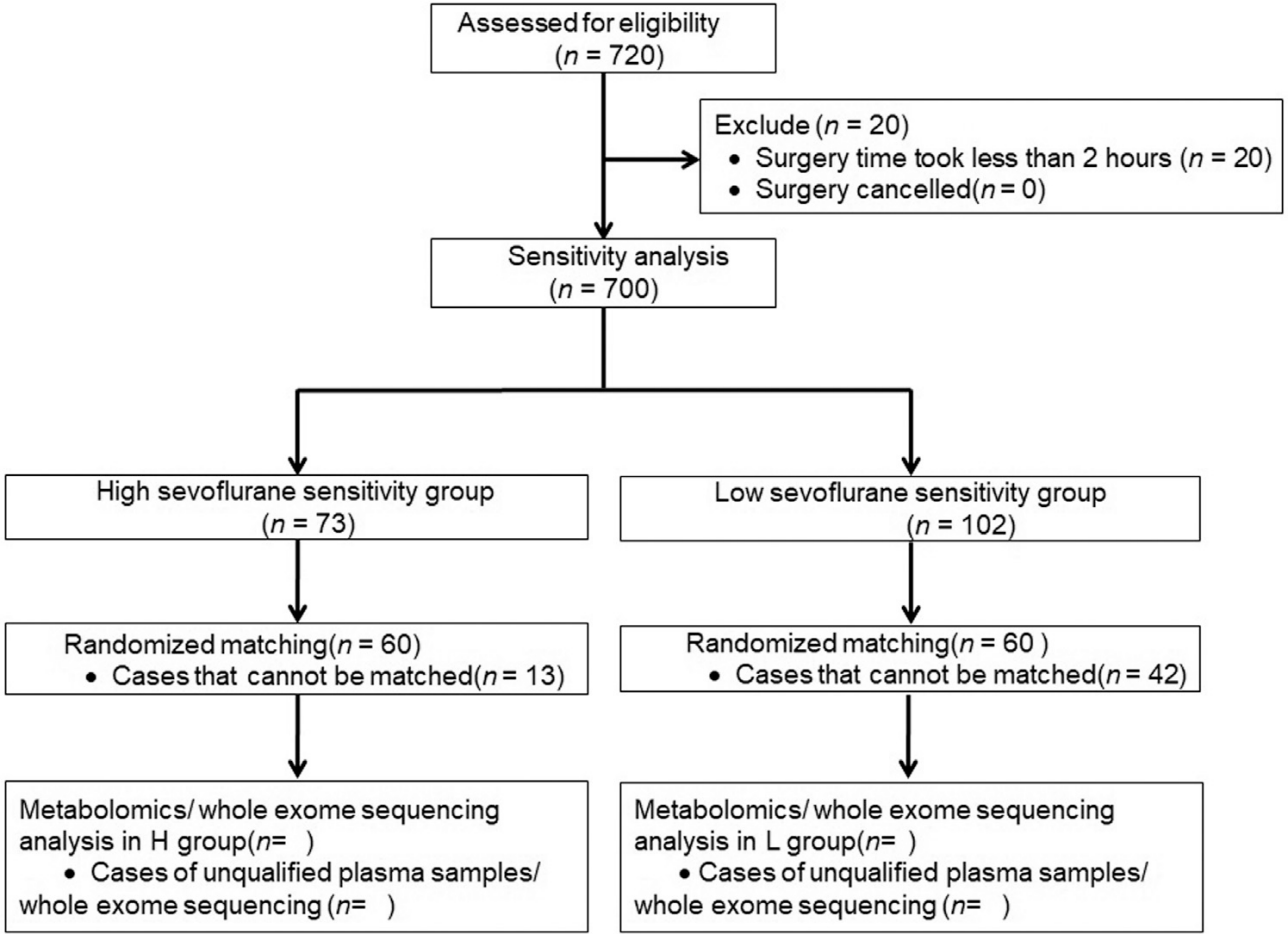
Flow diagram of the study.

This prospective, single-centre, cohort study protocol (ChiCTR-1800014327) has been approved by the Ethical Committee of West China Hospital of Sichuan University. The flow diagram of this study is shown in Figure 1. 720 patients undergone radical gastrectomy and colorectal surgery were recruited in the study between January 2018 and September 2018 at West China Hospital of Sichuan University. No preoperative medication was given. Anesthesia was induced with inhaled sevoflurane, a bolus of sufentanil (0.2–0.4 μg/kg) and *cis*-atracurium (0.2–0.3 mg/kg). After endotracheal intubation, ET_sevo_ was adjusted according to vital signs by turning the volatile tank scale up or down (0.5–1%) to maintain the BIS value at 40–60 with continuous infusion of sufentanilat a rate of 0.1–0.2 μg/kg/h. Patients were given a bolus of *cis*-atracurium and additional sufentanil according to clinical requirements for anesthesia maintenance. Three milliliters of venous blood were collected before anesthesia induction or at 2 h after the beginning of surgery. The blood sample was then centrifuged and the plasma was extracted and stored at −80°C for subsequent plasma metabolomics. Four milliliters of venous blood were collected before anesthesia induction and stored at −80°C, and nucleated cells were selected for whole-exome sequencing.

The mean ETsevo from 20 min after endotracheal intubation to 2 h after starting surgery (steady state) was calculated in all patients. The data with normal distribution was present with mean ± standard deviation (SD). High-sensitivity was defined by an ET_sevo_ at least 1 standard deviation lower than the mean. Low-sensitivity was defined by an ET_sevo_at least 1 standard deviation higher than the mean. We have paired patients from the high-sensitivity group (group H) and low-sensitivity group (group L) according to age, sex, operation method (laparotomy/laparoscopic surgery), body mass index (BMI), *ASA* physical status classification, vital signs, end-tidal partial pressure of carbon dioxide (P_ET_CO_2_), BIS values, ephedrine use, and the sufentanil and *cis*-atracurium doses at anesthesia induction and steady state. We performed follow up to determine the occurrence of intraoperative awareness, postoperative nausea and vomiting in patients at the post-anesthesia care unit, and at postoperative day 1 and 3.

The plasma collected before anesthesia induction and at 2 h after starting surgery in group H and group L was labelled with group H-before, H-after, L-before and L-after, respectively. Quality control of all plasma samples were implemented. The stability of the instrument and the acquired signal were monitored in real-time to ensure the quality of process (process quality control). The chromatography mass spectrometry may be affected by the aging circuit board, humidity, temperature, vibration and other factors, which will cause the fluctuation of acquired signal. Therefore, data processing is required to obtain high quality data (data quality control).

LC-MS/MS analysis will be performed using an UHPLC system (1,290, Agilent Technologies) coupled to a Q Exactive Orbitrap high-resolution mass spectrometer. The mobile phase composition was divided into positive and negative ionic modes. Each mode included mobile phase A and mobile phase B. Mobile phase setting: 0.1% formic acid and 5 mmol/L ammonium acetate were the mobile phases of positive and negative ion mode A; and acetonitrile were the mobile phases of positive and negative ion mode B. ProteoWizard software was used to transform the original data into mzML format. XCMS software was used to correct peak recognition, peak extraction, peak alignment and peak integration. OSI-SMMS software was used to identify the differential metabolites (Version 1.0, Dalian Dataso Information Technology Co., LTD.).

The differential metabolites were mapped to KEGG metabolic pathways by enrichment analysis. Several differential metabolites will be selected according to the aims of the study. Multiple linear regression analysis will be conducted using MetaboAnalyst 4.0 software to explore the correlation between the level of differential metabolites and ET_sevo_ and to calculate the area under the concentration-time curve of ET_sevo_ (AUC_sevo_).

Genomic DNA in group H and group L will be extracted and subjected to sample quality control. The OD value of nucleic acid was detected by nanodrop microspectro photometer. A260/A280 ratio <1.8 indicates protein pollution. A260/A280 ratio >2.0 indicates RNA pollution. The intact genomic DNA was present as a single band after agarose gel electrophoresis. Genomic DNA was processed after quality control, including ultrasonic fragmentation, DNA end repair and sequencing adapter ligation at both ends. Exons will be captured on the liquid phase platform. After capturing fragments using PCR amplification, sequencing will be performed using the Illumina HiSeq 4,000 platform. The raw data will be analysed for quality control. High quality clean reads were obtained by strict filtration. The steps of filtering are: 1) remove adapter and retain remaining reads; 2) remove reads containing more than 10% of N; 3) remove low-quality reads (the number of bases with mass value Q ≤ 20 accounts for more than 50% of the whole read). The comparison software BWA (0.7.15) and MEM algorithm were used to compare the filtered reads to the reference genome. Then, the SAM format comparison results were obtained, and the SAM tool was used to output the comparison results as BAM format files. Finally, Picard 1.129 software (Mark Duplicates) was used to mark duplicate reads, and the depth and coverage of labelled reads were calculated. Allele frequency screening was used to find SNPs. The variations in SNPs will be analysed using comparative statistics with reference genomes. ANNOVAR software will be used for advanced annotation of detected variations. Then GWAS will be used to investigate the correlation between SNPs and ETsevo in group H and group L. After different models of GWAS analysis was completed, the distribution of actual *p*-values and theoretical *p*-values under different models will be compared by Q-Q diagram to determine the optimal model. Bonferroni multiple test correction method will be used to determine the significance threshold of *p* value and screen the SNPs in the region of strongest correlation for subsequent analysis. The results of GWAS analysis will be presented in the form of Manhattan Plot. The screening criteria for SNPs includes: 1) *p* < 0.001; 2) non-synonymous mutation; 3) from the exon region; 4) mutant genes with known functions; and 5) the difference in SNP mutation frequency between group H and group L was more than 5%.

### Interventions

Bradycardia is defined as a HR of <60 beats per minute. If HR is below 50 beats per minute with hypotension, atropine or ephedrine will be administered. Tachycardia is defined as a HR > 100 beats per minute. If the depth of anesthesia and the degree of analgesia are sufficient and a HR > 90 beats per minute is observed, esmolol will be administered. Hypotension is defined as systolic pressure <90 mmHg or diastolic pressure <50 mmHg. Hypertension is defined as over 20% of the baseline blood pressure. If hypotension or hypertension occurs, metaraminol, urapidil hydrochloride or nicardipine hydrochloride will be administered.

### Objectives

The present study aims are:

1) To screen differential sensitivity to sevoflurane in patients;
2) To find differential metabolites in group H compared with group L using plasma metabolomics;
3) To find differential SNPs and genes in group H compared with group L using WES, GWAS and bioinformatics.

### Outcomes

The primary outcome is the incidence of high sensitivity to sevoflurane in patients. Secondary outcomes include the following:

1) The average ETsevo at stable state;
2) Tracheal extubation time;
3) Incidence of intraoperative awareness;
4) The use of vasoactive drugs;
5) Incidence of postoperative nausea and vomiting.

### Inclusion Criteria

Consenting patients will be eligible if.

1) Aged 18–65 years;
2) Undergoing radical gastrectomy or colorectal surgery;
3) In American Society of Anesthesiologists (ASA) category 1–2;
4) Operation time duration exceeding 2 h;
5) Written informed consent provided.

### Exclusion Criteria

Patients will be excluded if any of the following exists:

1) Allergy to volatile anesthetics;
2) Cerebrovascular diseases, including cerebral infarction, transient cerebral ischemia, intracranial hematoma, cerebrovascular malformation, and cerebral hemorrhage;
3) Cardiovascular diseases, including coronary heart disease, arrhythmia, heart failure, valvular disease, and congenital heart disease;
4) Alcohol addiction;
5) Malignant hyperthermia;
6) Abnormal liver or renal function.

### Sample Size

In this study, the sample size was calculated based on our preliminary experimental results. We estimate the incidence of high-sensitivity patients as 12%, with a power of 80% and *α* = 0.05 (two sided); thus, we need 329 patients in each group. Assuming a drop-out rate of 4% during the study, 342 patients per group are required. Therefore, a total of 700 patients are required in our study.

### Statistical Methods

Statistical analyses will be performed using SPSS (Version 22.0, IBM Corp., Armonk, NY, United States). Normally distributed data will be presented as the mean ± SD, whereas non-normally distributed data will be presented as the median and interquartile range. Analysis of variance will be used for continuous variables. Categorical data will be presented as numbers and compared using the chi-squared test. Groups will be compared using Student’s *t*-test (normally distributed data) or the Mann-Whitney *U* test (nonnormally distributed data). After identification of differential metabolites and multivariate statistical analysis, metabolites with significant differences between groups will be screened by variable importance in projection (VIP) of model variables with orthogonal partial least squares discriminant analysis (OPLS-DA) and the *p* value of the *t* test for univariate statistical analysis. Metabolites with *p* < 0.05 and VIP ≥1 were considered differential metabolites between comparisons. To identify correlations between metabolite levels and the amount of sevoflurane exposure, multivariate linear regression or Pearson’s correlation analysis will be performed using MetaboAnalyst 4.0 software.

## Discussion

The sensitivity to volatile anesthetics may be related to postoperative outcomes of patients and the action mechanism of volatile anesthetics. Sevoflurane is widely used for various surgeries. However, very few studies described differential sensitivity to sevoflurane among patients. We previously found that the genes associated with mitochondrial energy metabolism were involved in sensitivity to sevoflurane in *Drosophila*, and changes in these genes can cause changes in the levels of metabolites in the body (Liu et al., 2009). In clinic, we found that the end-tidal sevoflurane concentration (ETsevo) was different among patients at the same depth of anesthesia. However, the precise underlying mechanism remains unclear. In addition, genetic variations in *Drosophila*, nematodes and mice affect their sensitivity to volatile anesthetics (Morgan and Sedensky, 1994; Kayser et al., 1999; Falk et al., 2006; Borghese et al., 2012; Hu et al., 2012; Zalucki et al., 2015). Therefore, changes in metabolites and genetic variations may be involved in anesthetic efficacy (Olufs et al., 2018). However, no current study has demonstrated whether changes in metabolites and genetic variations are associated with anesthesia sensitivity in patients.

This study is a prospective observational study to identify potential metabolites, SNPs and genes related to sevoflurane sensitivity. Cases with different levels of sevoflurane sensitivity will be screened according to the ETsevo for maintaining the target depth of anesthesia. After pairing cases of high-sensitivity and low-sensitivity to sevoflurane, differential metabolites, SNPs and genes may be identified through plasma metabolomics, whole-exome sequencing (WES), GWAS and bioinformatics. Therefore, this study will identify potential markers of sevoflurane sensitivity for further studies.

There are several limitations in this study. First, cerebral spinal fluid metabolomics were not examined, which may be more relevant to anesthesia sensitivity. Second, the sample size may be small to fully explain the sensitivity to sevoflurane. However, in future studies, we can further expand the sample size and include other surgical patients to demonstrate differential sensitivity of patients to sevoflurane. Furthermore, we cannot exclude the effects of surgery on our results. In addition, this study does not validate the effects of the identified metabolites, SNPs and genes through clinical studies or experiments on animals, which needs a further confirmation in future.

Currently, limited studies have described patient sensitivity to sevoflurane. Evidence from this prospective observational study may help to provide a scientific basis for the sensitivity of patients to sevoflurane and the mechanism of action of volatile anesthetics. The study provides an opportunity to investigate the different sensitivity of patients to sevoflurane and to explore the underlying mechanism.

## Data Availability

The original contributions presented in the study are included in the article/Supplementary Material, further inquiries can be directed to the corresponding author.
